# Clinical study of ^18^F-FDG PET/CT radiomics in differentiating pulmonary solitary solid adenocarcinoma nodules and inflammatory nodules

**DOI:** 10.7717/peerj.21188

**Published:** 2026-06-01

**Authors:** Yi fan Liao, Song Zhang, Bao yu Wan, Jia xu Li, Jie Deng, Jie Hu, Xian li Qin

**Affiliations:** Department of Nuclear Medicine, Xinqiao Hospital, Army Medical University, ChongQing, China

**Keywords:** Lung adenocarcinoma, Pulmonary nodule, Radiomics, Fluorodeoxyglucose, Positron emission tomography-computed tomography

## Abstract

**Objective:**

This study aimed to assess the diagnostic value of ^18^F-FDG PET/CT radiomics in distinguishing adenocarcinoma from inflammatory lesions in pulmonary solitary solid nodules (solid pulmonary nodules).

**Methods:**

A total of 222 patients with Solid pulmonary nodules were retrospectively analyzed and randomly divided into two groups: a training set (*n* = 155) and a validation set (*n* = 67). Radiomic features were extracted from positron emission tomography/computed tomography (PET/CT) images, and optimal features were selected from the training set. Three model groups were created (CT, PET, and PET+CT) using six machine learning classifiers: Support Vector Machine (SVM), Random Forest (RF), Stochastic Gradient Descent (SGD), K-Nearest Neighbors (KNN), Extreme Gradient Boosting (XGBoost), and Light Gradient Boosting Machine (LightGBM). The performance of the models was evaluated using the area under the receiver operating characteristic curve (ROC).

**Results:**

A total of eleven, nine, and fourteen optimal features were identified for the CT, PET, and PET+CT groups, respectively. In the validation set, the Area Under the Curve (AUC) values for the CT models ranged from 0.731 to 0.831, for the PET models from 0.746 to 0.810, and for the PET+CT models from 0.800 to 0.847. Among these, the PET+CT model developed using the Random Forest (RF) classifier demonstrated the best diagnostic performance, with an AUC of 0.847, sensitivity of 0.804, and specificity of 0.821. Decision curve analysis (DCA) confirmed that the model has favorable clinical utility, while calibration curves showed a good agreement between predicted and observed outcomes.

**Conclusion:**

The PET+CT radiomics models outperformed the single-modality models in distinguishing Solid pulmonary nodules adenocarcinoma from inflammatory lesions. Overall, the RF-based PET+CT model achieved the highest diagnostic efficacy and indicates promising potential for clinical application.

## Introduction

Lung cancer is the leading cause of cancer-related mortality worldwide, and despite a decline in mortality rates, it continues to result in more deaths than colorectal, breast, and prostate cancers combined (Filho et al., 2025; Ge et al., 2023; Siegel et al., 2025). Lung adenocarcinoma, the most common histological subtype of lung cancer, is responsible for approximately 50% of lung cancer-related deaths. Despite recent advancements in treatment strategies, the long-term survival outcomes for patients with lung adenocarcinoma remain suboptimal (Kara et al., 2021; Wei et al., 2023). With the widespread use of computed tomography (CT), there is an increasing detection of pulmonary solitary nodules. However, determining the nature of these nodules can be challenging. Pulmonary solitary solid nodules (solid pulmonary nodules) have a higher percentage of benign lesions compared to ground-glass opacities and partially solid nodules, complicating the early diagnosis of lung adenocarcinoma (Ning et al., 2023). On the other hand, malignant pulmonary nodules are closely associated with a higher mortality risk and poorer prognosis, making it essential to accurately differentiate between benign and malignant nodules for effective treatment planning (Sun et al., 2021). We focused on solitary solid pulmonary nodules, defined as lesions that completely obscure the underlying lung parenchyma on CT, and distinct from subsolid nodules (pure ground-glass or part-solid nodules). Subsolid nodules represent a different radiologic–pathologic spectrum and management pathway; therefore, they were not included in this study.

Conventional imaging assessment relies primarily on morphological features such as size, margin characteristics, shape, and location (McWilliams et al., 2013). However, substantial overlap exists between benign and malignant nodules, limiting the diagnostic value of these features. Functional imaging techniques have been introduced to improve discrimination, including positron emission tomography (PET). [^1^^8^F]Fluorodeoxyglucose (^1^^8^F-FDG) positron emission tomography/computed tomography (PET/CT) provides not only anatomical details but also functional information by reflecting glucose metabolism within lesions, thus offering an indirect marker of malignancy (Mehta et al., 2024). This modality has been widely adopted for qualitative diagnosis and preoperative evaluation of pulmonary nodules (Lucia et al., 2024). Prior studies have reported variable diagnostic performance for morphology-based CT assessment of pulmonary nodules, and qualitative ^18^F-FDG PET/CT interpretation may suffer from false positives in inflammatory/granulomatous disease and false negatives in certain low-FDG-avid adenocarcinomas, resulting in overlapping imaging appearances. Therefore, additional quantitative approaches such as radiomics may improve discrimination (Iskender et al., 2012). While histopathological examination remains the gold standard for diagnosing indeterminate pulmonary nodules (Na et al., 2020), it is an invasive procedure that can take a long time for results.

Radiomics has emerged as a promising, non-invasive approach that overcomes some of these limitations. By applying advanced mathematical and statistical algorithms, radiomics enables the high-throughput extraction of quantitative features from regions of interest (ROIs), capturing subtle spatial, textural, and intensity patterns that are imperceptible to the human eye (Mayerhoefer et al., 2020; Scapicchio et al., 2021). These features can then be integrated with machine learning algorithms to build predictive models, potentially improving diagnostic accuracy and supporting clinical decision-making. In pulmonary nodule assessment, radiomics has been increasingly used to quantify lesion heterogeneity beyond visual evaluation and to support discrimination between benign and malignant nodules. Prior studies have reported that CT- or PET/CT-based radiomic signatures, combined with machine-learning classifiers, may improve diagnostic performance compared with conventional qualitative interpretation. However, reported results vary across nodule types, imaging protocols, and modeling strategies, and evidence specifically focusing on solid pulmonary nodules remains limited (Wang et al., 2025b; Zhang et al., 2025b).

In this study, we extracted radiomic features from ^1^^8^F-FDG PET/CT images and constructed multiple machine learning models to classify Solid pulmonary nodules as adenocarcinoma or inflammatory lesions. We further evaluated the diagnostic performance of different models and sought to determine the optimal approach. Specifically, we aimed to develop a decision-support model to assist clinicians in managing indeterminate solid pulmonary nodules on PET/CT, potentially informing choices between tissue confirmation (biopsy/surgery) and short-interval imaging follow-up within a multidisciplinary setting.

## Methods

### Patients

This retrospective study included patients with solid pulmonary nodules who underwent whole-body ^18^F-FDG PET/CT examinations at the Department of Nuclear Medicine. This study was conducted in accordance with the principles of the Declaration of Helsinki. Ethical approval was obtained from the Medical Ethics Committee of the Second Affiliated Hospital of Army Medical University (Approval No. 2024-yan-NO.081-01). Given the retrospective design of this study and the use of anonymized patient data, the requirement for individual informed consent was waived by the ethics committee. Histopathological confirmation was obtained for all patients through either surgical resection or biopsy, serving as the reference standard. Inclusion criteria were as follows: (1) CT findings showing a solitary solid pulmonary nodule with a maximum diameter of ≤ three cm without any ground-glass component; and (2) availability of postoperative or biopsy-based histopathological results. Exclusion criteria included: (1) multiple pulmonary lesions (≥ 2 primary nodules); (2) absence of FDG uptake on PET images; (3) lesions unsuitable for accurate region of interest (ROI) delineation; (4) poor image quality due to motion or metallic artifacts affecting feature extraction; and (5) history of malignancy. The final cohort (*n* = 222) was randomly divided into a training set (*n* = 155) and a validation set (*n* = 67) at an approximate 7:3 ratio using stratified sampling by pathology, ensuring similar class proportions in both sets.

### PET/CT image acquisition

All patients fasted for at least 4–6 h and avoided strenuous exercise before examination. Height, body weight, and fasting blood glucose (<11.1 mmol/L) were measured. ^18^F-FDG (Chongqing Atom High-Tech Pharmaceutical Co., Ltd.; chemical purity >95%) was intravenously injected at a dose of 3.7–5.55 MBq/kg. Patients rested quietly for 40–60 min post-injection, during which urination was allowed. Prior to scanning, patients were instructed to drink 500 ml of water and void urine. PET/CT scans were performed using a Philips GEMINI TF TOF 64 scanner in the supine position, covering from the skull vertex to the upper third of the femur. CT scans were first acquired with parameters: 120 kV, 150 mAs, pitch 0.875, and slice thickness three mm. Subsequently, PET images were obtained in 3D mode with 7–10 bed positions, 2 min/bed position. PET data were corrected for CT-based attenuation and reconstructed using the ordered subset expectation maximization (OSEM) algorithm. Fused PET/CT images were then generated for further analysis.

### Tumor segmentation and feature extraction

Radiomics feature extraction was performed using LIFEx software (version 7.3.0, https://www.lifexsoft.org/) on both PET and CT images. For CT images, regions of interest (ROIs) were manually delineated slice by slice, while for PET images, ROIs were semi-automatically segmented using 40% of the maximum standardized uptake value (SUVmax) as the optimized threshold. In addition, conventional PET/CT parameters, including maximum lesion diameter, SUVmax, metabolic tumor volume (MTV), and total lesion glycolysis (TLG), were also collected using the same software. All ROI delineations were initially performed by a nuclear medicine physician with more than five years of diagnostic experience, and subsequently reviewed and confirmed by a senior physician with over ten years of professional experience.

### Feature selection

**Figure 1:**
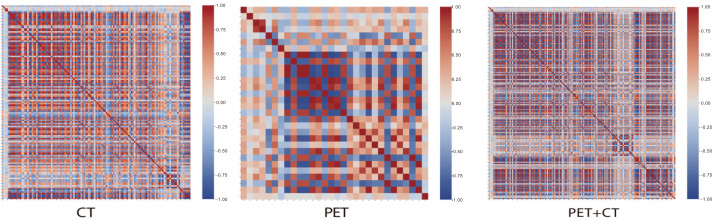
Correlation heatmap of radiomics features used in the PET+CT model.

Radiomics feature selection and dimensionality reduction were conducted in several steps. First, all extracted features were standardized, and those with near-zero variance were removed. Then, univariate statistical tests were applied: normally distributed features with equal variances were compared using the *t* test, while others were evaluated with the Mann–Whitney U test, and only features with significant differences (*P* < 0.05) were retained. Next, Pearson correlation analysis was performed to eliminate redundant features with high collinearity, and one feature from each highly correlated pair (—r— > 0.90) was removed to mitigate multicollinearity; the feature with stronger univariate association and/or clearer interpretability was retained. Finally, the least absolute shrinkage and selection operator (LASSO) regression combined with 10-fold cross-validation was used to further reduce dimensionality and identify the most informative radiomics features, which were subsequently used to construct the machine learning models. To assess redundancy among extracted features, we calculated the pairwise correlations. As shown in Fig. 1, radiomic features exhibit substantial pairwise correlations in CT, PET, and PET+CT feature sets, indicating potential redundancy and multicollinearity if all features are entered into modeling simultaneously. This supports the necessity of correlation-based redundancy reduction prior to model fitting, improving model stability and reducing overfitting risk.

**Figure 2:**
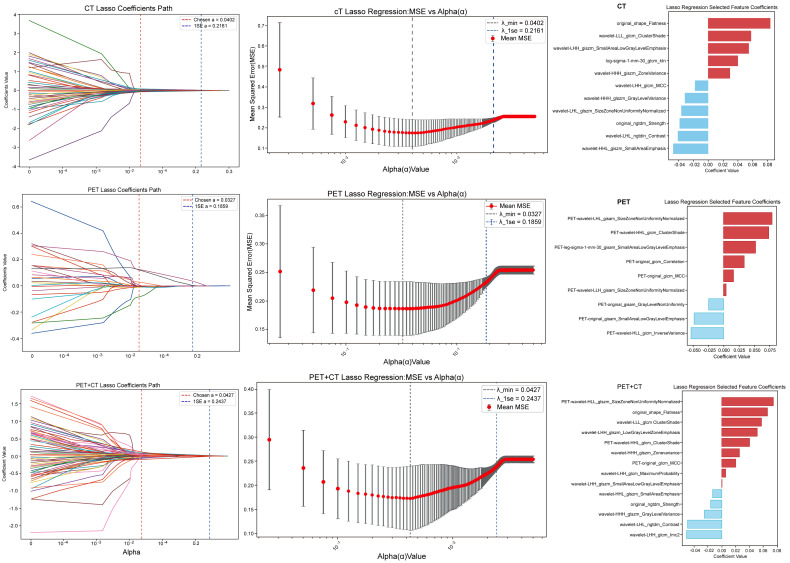
Bar plot of radiomic features and their corresponding coefficients selected by LASSO regression.

Figure 2 demonstrates the LASSO regularization process for each modality: the coefficient trajectories shrink toward zero as α increases, while the cross-validated MSE curve identifies an optimal penalty that balances model complexity and predictive error. The resulting non-zero–coefficient features and their coefficient directions summarize the final radiomic signatures used for downstream machine-learning classifiers. These preprocessing and selection steps were intended to improve feature stability and reduce overfitting. However, formal robustness analyses, including interobserver reproducibility assessment, test–retest evaluation, and external validation across scanners and protocols, were not comprehensively performed in the present study.

### Model construction and evaluation

Radiomics-based predictive models were developed using six machine learning classifiers: support vector machine (SVM), random forest (RF), stochastic gradient descent (SGD), K-nearest neighbors (KNN), extreme gradient boosting (XGBoost), and light gradient boosting machine (LightGBM). The optimal radiomics features derived from the training set were used as inputs to each classifier. Model training was performed on the training set, while independent evaluation was carried out on the validation set to ensure generalizability.

Model performance was assessed using accuracy, sensitivity, specificity, F1-score, and the area under the receiver operating characteristic curve (AUC), with the validation set AUC regarded as the primary indicator of diagnostic efficacy. In addition, decision curve analysis (DCA) was performed to evaluate the net clinical benefit across a range of threshold probabilities, and calibration curves were plotted to examine the agreement between predicted and observed outcomes in both the training and validation sets. In the present study, the model output was expressed as the predicted probability of malignancy for each lesion. A higher predicted probability indicates a greater model-estimated likelihood of adenocarcinoma rather than inflammatory nodules within the study setting.

### Statistical analysis

All data processing was performed using Python (version 3.7), and statistical analyses were conducted with SPSS software (version 23.0). The Kolmogorov–Smirnov test was used to assess the normality of continuous variables. Normally distributed data were expressed as mean ± standard deviation (SD), and group comparisons were conducted using the independent-samples *t* test. Non-normally distributed data were expressed as median (interquartile range, IQR), and intergroup comparisons were performed using the Mann–Whitney U test. Categorical variables were presented as frequencies and percentages, and compared between groups using the chi-square test. A two-tailed *P* value < 0.05 was considered statistically significant.

## Result

### Baseline characteristics

A total of 222 patients with solid pulmonary nodules were included in the study, with 155 assigned to the training set and 67 to the validation set. Pathological diagnoses comprised 129 cases of lung adenocarcinoma and 93 cases of inflammatory nodules. Baseline demographic, pathological, and imaging characteristics were well balanced between the two sets, with no statistically significant differences observed (all *P* > 0.05), as summarized in Table 1. Table 2 presents logistic regression results for clinical characteristics. In multivariable analysis, sex, age, SUVmax, and maximum nodule diameter remained independent predictors of adenocarcinoma (all *P* < 0.05), whereas TLG and MTV were not significant after adjustment. In AUC analysis, SUVmax showed the best univariate discrimination (AUC = 0.692, 95% CI [0.622–0.761]), and the clinical-only multivariable model achieved an AUC of 0.768 (95% CI [0.706–0.830]), serving as a baseline comparator for radiomics models.

**Table 1:** Baseline characteristics of patients in the training and validation sets.

Variables	Over all (*n* = 222)^1^	Training set (*n* = 155)^1^	Validation set (*n* = 67)^1^	*p* value^2^
Pathology				>0.999
Inflammatory	93.0 (41.9%)	65.0 (41.9%)	28.0 (41.8%)	
Adenocarcinoma	129.0 (58.1%)	90.0 (58.1%)	39.0 (58.2%)	
Sex				0.166
Female	89.0 (40.1%)	57.0 (36.8%)	32.0 (47.8%)	
Male	133.0 (59.9%)	98.0 (63.2%)	35.0 (52.2%)	
Age	57.0 [51.0, 65.0]	57.0 [51.0, 63.0]	58.0 [50.0, 66.0]	0.337
Max nodule diameter	2.0 [1.6, 2.4]	2.0 [1.7, 2.4]	2.0 [1.6, 2.4]	0.832
SUVmax	3.9 [2.5, 6.4]	3.9 [2.5, 6.8]	4.0 [2.7, 5.8]	0.932
MTV	3.0 [2.0, 5.0]	3.0 [2.0, 6.0]	3.0 [2.0, 5.0]	0.259
TLG	8.0 [4.0, 17.0]	9.0 [4.0, 18.0]	7.0 [4.0, 15.0]	0.376

**Notes.**

1 n (%); Median [Q1, Q3].

2 Pearson’s Chi-squared test; Kruskal–Wallis rank sum test.

**Table 2:** Results of logistic regression analysis for clinical characteristics.

Characteristic	Univariate analysis			Multivariable analysis		
	OR	95% CI	*P*-value	OR	95% CI	*P*-value
Sex	2.08	1.19–3.69	0.011^*^	2.23	1.2–4.2	0.012^*^
Age	1.05	1.02–1.08	<0.001^*^	1.05	1.02–1.09	0.001^*^
TLG	1.04	1.02–1.08	0.003^*^	0.97	0.92–1.01	0.119
MTV	1.02	0.92–1.13	0.748			
SUVmax	1.35	1.19–1.56	<0.001^*^	1.42	1.19–1.72	<0.001^*^
Max nodule diameter	2.85	1.68–4.95	<0.001^*^	2.24	1.08–4.76	0.032^*^

**Notes.**

* Represents *P* < 0.05.

### Comparison between different models

Table 3 summarizes the performance of the PET+CT radiomics models, while the detailed results of the CT-only and PET-only models across all classifiers are reported in Table S1. Across all three groups, the PET+CT radiomics models consistently outperformed the CT- and PET-only models in nearly all evaluation metrics. The CT-based models achieved validation AUCs ranging from 0.731 to 0.831, with the XGBoost classifier showing the best performance within this group (AUC = 0.831). The PET-based models generally yielded lower validation AUCs (0.746–0.807), with the RF classifier performing best among the six machine learning algorithms (AUC = 0.810). In contrast, the PET+CT combined models demonstrated superior discrimination, achieving higher validation AUCs across all classifiers (0.800–0.847), with the RF classifier again showing the highest diagnostic performance (AUC = 0.847, sensitivity = 0.804, specificity = 0.821). Notably, the PET+CT models also showed improved F1-scores and balanced sensitivity–specificity profiles, suggesting better generalization and robustness compared to single-modality models. These findings indicate that the integration of PET and CT radiomic features can complement one another, enhance model performance, and provide more reliable diagnostic information for differentiating pulmonary solitary solid adenocarcinomas from inflammatory nodules. The detailed performance metrics for each classifier are provided in Table S1.

**Table 3:** Summary of performance metrics for PET+CT models on training and validation sets.

Model	Set	AUC (95% CI)	Accuracy	Sensitivity	Specificity	F1-score
SVM	Training	0.881 (0.828–0.932)	0.845	0.789	0.677	0.824
	Validation	0.843 (0.765–0.921)	0.746	0.893	0.806	0.712
RF	Training	0.849 (0.799–0.892)	0.781	0.746	0.75	0.734
	Validation	0.847 (0.775–0.919)	0.791	0.804	0.821	0.75
SGD	Training	0.891 (0.856–0.926)	0.832	0.82	0.667	0.794
	Validation	0.838 (0.785–0.891)	0.716	0.84	0.75	0.655
KNN	Training	0.833 (0.777–0.888)	0.761	0.694	0.679	0.73
	Validation	0.800 (0.716–0.885)	0.731	0.819	0.769	0.679
XGBoost	Training	0.831 (0.764–0.897)	0.768	0.716	0.778	0.727
	Validation	0.843 (0.742–0.945)	0.806	0.807	0.825	0.764
LightGBM	Training	0.810 (0.742–0.879)	0.729	0.677	0.71	0.677
	Validation	0.843 (0.733–0.954)	0.776	0.767	0.833	0.746

**Figure 3:**
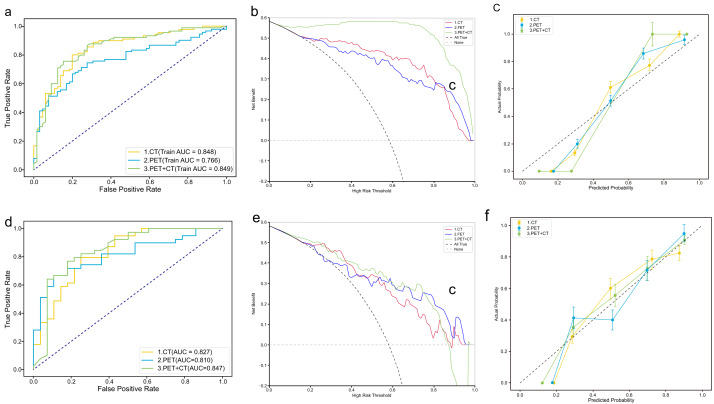
Performance comparison of CT, PET, and combined PET+CT radiomics models across training and validation sets.

**Figure 4:**
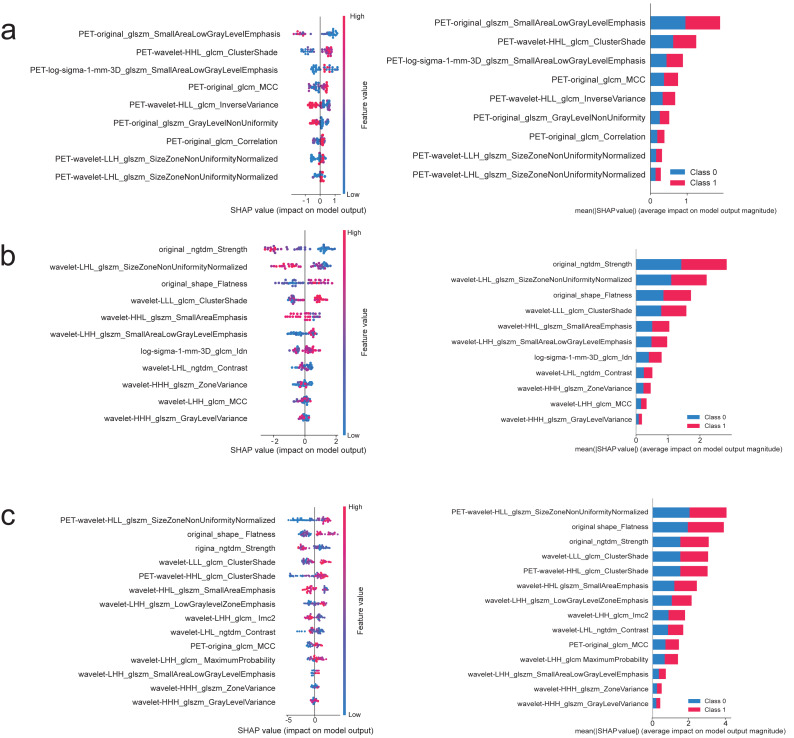
SHAP summary and feature-importance plots for radiomics models built on PET (A), CT (B), and combined PET+CT (C) modalities. Positive SHAP values indicate increased predicted probability of adenocarcinoma (Class 1).

Decision curve analysis (DCA) showed that all three models provided a higher net clinical benefit than the “treat-all” and “treat-none” strategies across most threshold probabilities, with the PET+CT model consistently achieving the greatest net benefit. Figure 3 compares the ROC performance of CT-, PET-, and PET+CT-based models. Overall, the PET+CT models showed competitive and generally more stable performance across classifiers, supporting the added value of multimodal integration. Within the PET+CT group, the RF model achieved the highest validation AUC (0.847). SHAP plots were used to visualize and compare feature contributions across different models (Fig. 4).

## Discussion

In this study, we evaluated the diagnostic performance of ^18^F-FDG PET/CT radiomics in distinguishing pulmonary solitary solid adenocarcinoma nodules from inflammatory nodules using six machine learning classifiers. Our findings demonstrated that PET+CT-based radiomics models consistently outperformed CT- and PET-only models across multiple performance metrics, including AUC, accuracy, sensitivity, specificity, and F1-score. Among all classifiers, the Random Forest model exhibited the highest validation AUC (0.847) in the PET+CT group, suggesting comparatively stable performance within the validation set.

The management of solid pulmonary nodules is highly dependent on accurate differentiation between benign and malignant etiologies. Inflammatory nodules should be spared from unnecessary interventions, whereas malignant nodules require timely and aggressive treatment strategies (Owens et al., 2023; Sun et al., 2023; Tatcı et al., 2024). Inflammatory nodules should avoid unnecessary interventions, while cancerous nodules require prompt, aggressive, and effective treatment. Although there are typical morphological features that can help distinguish between benign and malignant nodules, some benign lesions may present CT images that resemble malignant tumors. With the increasing use of ^18^F-FDG PET/CT in clinical practice, certain benign granulomatous inflammations, such as tuberculosis and both acute and chronic inflammations, can be misidentified as neoplastic lesions due to their elevated FDG metabolism. Conversely, some malignant lesions, such as mucinous well-differentiated adenocarcinomas, may show low FDG uptake, leading to potential misdiagnosis as well (Wang et al., 2025a; Zhang et al., 2025a). Radiomics allows for the conversion of images into high-throughput data, revealing functional and molecular-level metabolic changes that are not easily detected through visual inspection. This approach enhances reproducibility and reduces diagnostic variability that can arise from individual physician experience (Anan, Zainon & Tamal, 2022; Chang et al., 2022; Nakajo et al., 2024; Yu et al., 2024). Several studies have explored PET/CT-based radiomics for differentiating benign from malignant solitary pulmonary lesions or pulmonary nodules using machine learning approaches. However, published cohorts often include mixed lesion types and nodule components, and the optimal classifier and imaging modality remain inconsistent across studies. Zhang et al. (2025c) developed dual-phase ^18^F-FDG PET/CT radiomics models (combining CT with routine and delayed PET features) in 132 solitary pulmonary lesions and found that the CT+(PET2–PET1)/PET1 model achieved the best discrimination between benign and malignant lesions (AUC ≈ 0.898). Tian et al. (2024) retrospectively analyzed ^18^F-FDG PET/CT scans from 228 patients (adenocarcinoma *vs* pulmonary granulomas), extracted radiomic features from intranodular and perinodular regions, and showed that combining intra- and perinodular features (within ∼2-voxel margins) improved diagnostic performance compared with intranodular features alone.

In recent years, researchers have used radiomics to distinguish between benign and malignant pulmonary nodules. Although promising diagnostic models have been developed, the variety of nodule types and the lack of pathological differentiation during model construction have affected the models’ stability. This study utilizes only pathologically confirmed pulmonary nodules for modeling, which enhances the study’s credibility and validation effectiveness. Current literature reports PET/CT radiomics models for differentiating solitary pulmonary nodules; however, there is no consensus on which classifier to use (Shen et al., 2021; Zhou et al., 2021). Choosing the right model involves complex considerations, such as screening methods, the number of features, and validation techniques. Therefore, while aiming for optimal model performance, the modeling process should also prioritize ease of implementation, even if this may lead to some trade-offs in performance (Demircioğlu, 2022).

In our results, the XGBoost classifier yielded the highest validation AUC (0.831) among CT-based models, aligning with the findings of Selvam et al. (2023). In the PET group, the RF classifier achieved the best performance (AUC = 0.810), suggesting that modeling outcomes are strongly influenced by classifier choice and modality. Notably, model ranking depends on the evaluation metric. While XGBoost achieved higher values in some threshold-dependent metrics (*e.g.*, accuracy and F1-score) and SVM provided the highest sensitivity at the selected operating point, we prioritized validation AUC as our primary endpoint because it is threshold-independent and better captures overall discrimination across potential clinical thresholds. In contrast, accuracy/sensitivity/specificity/F1 can change when the decision threshold is shifted and may be influenced by class proportions. Under this framework, the RF-based PET+CT model yielded the highest validation AUC (0.847) and a balanced sensitivity–specificity trade-off, suggesting robust generalizability for differentiating solitary solid pulmonary adenocarcinoma nodules from inflammatory nodules. Our results are further supported by Brian et al. (Huang et al., 2022), who observed superior performance of CT-based over PET-based models in similar tasks. This trend may stem from the inherently higher spatial resolution of CT, which offers more informative radiomic features, as reflected in our selection of 11 features from CT *versus* nine from PET. These findings support the notion that image resolution contributes to the richness of extracted cellular-level information (Chang et al., 2021). Interestingly, the SGD and KNN classifiers deviated from this pattern, performing better in the PET group than in the CT group. This variability is likely due to the limited sample size, especially in the validation set, which may introduce statistical fluctuations. In addition, this behavior may also reflect the algorithmic characteristics of these classifiers. SGD-based models (often linear classifiers trained *via* stochastic optimization) can be sensitive to feature scaling and hyperparameter settings (*e.g.*, learning rate and regularization), whereas KNN is a distance-based method influenced by the choice of *k* and is more susceptible to noise and local sample density. Moreover, PET-derived intensity features and CT texture features may exhibit different distributional properties, which can differentially affect the performance of SGD and KNN across modalities. Nevertheless, across all six classifiers, the combined PET+CT models consistently outperformed their CT-only and PET-only counterparts, highlighting the complementary value of metabolic and anatomical data. Notably, the RF classifier in the PET+CT group achieved the highest and most stable performance (AUCs of 0.849 and 0.847 in training and validation sets, respectively). Decision curve analysis demonstrated substantial clinical benefit, and calibration curves confirmed good model fitting.

Although the proposed PET/CT radiomics models demonstrated promising discriminatory performance in this cohort, these findings should be interpreted as exploratory rather than immediately clinically actionable. The present study provides preliminary evidence supporting the potential utility of radiomics for differentiating pulmonary solitary solid adenocarcinomas from inflammatory nodules, but the results are not sufficient to justify standalone clinical use at this stage. In practice, the model can be used after PET/CT acquisition and ROI delineation to generate a malignancy probability for solid pulmonary nodules. The output may assist risk stratification alongside radiologist interpretation and clinical factors, supporting decisions between early tissue confirmation for high-risk nodules and short-interval follow-up for lower-risk cases. However, because the model was developed in a retrospective single-center cohort without external validation, its operating characteristics should be interpreted cautiously until confirmed in broader populations. Thresholds can be adjusted according to clinical priorities and multidisciplinary consensus. The Delong test conducted in this study indicated a statistically significant difference between the CT group and the PET+CT group in the validation set for the KNN classifier (Z = −2.531, *P* < 0.05). However, no statistically significant differences were found among the validation sets for the other six machine learning classifiers (*P* ≥ 0.05). Although the RF classifier for the PET+CT group achieved the highest AUC value in the validation set, this difference was not statistically significant when compared to the models of the other groups. In real-world clinical practice, short-interval imaging follow-up is commonly recommended for indeterminate pulmonary nodules to monitor interval growth and evolving imaging characteristics, which may help differentiate inflammatory processes from malignancy. Major clinical guidelines, including those from the Fleischner Society and the American College of Chest Physicians (ACCP), advocate risk-adapted surveillance strategies, particularly for nodules with low-to-intermediate pretest probability of malignancy. In this context, radiomics-based risk estimation may complement follow-up strategies by facilitating early identification of patients who may benefit from prompt tissue confirmation, while supporting surveillance in lower-risk cases, thereby reducing unnecessary invasive procedures and optimizing clinical decision-making. Several factors may have contributed to these findings, including the single-center retrospective design and the limited sample size, particularly in the validation cohort. In addition, radiomic features may be susceptible to image noise and acquisition-related variability—especially when derived from the low-dose CT component of PET/CT acquired for attenuation correction/anatomical localization—which may differ from diagnostic chest CT in noise, spatial resolution, reconstruction parameters, and breathing conditions, thereby affecting feature stability and generalizability. Finally, inter- and intra-observer segmentation reproducibility was not quantitatively assessed (*e.g.*, ICC analysis), and future multicenter prospective studies will incorporate reproducibility testing and external validation, including validation on diagnostic chest CT across scanners/protocols. There was also a patient selection bias inherent in this retrospective study. Future work should focus on developing multimodal predictive frameworks that combine radiomic signatures with relevant clinical, laboratory, and pathological variables. Moreover, rigorous external validation across institutions using heterogeneous scanners, acquisition parameters, and reconstruction protocols will be crucial for determining whether the observed performance can be maintained under real-world conditions. These steps are necessary to improve the translational applicability of the present findings.

In summary, among the ^18^F-FDG PET/CT radiomics approaches for differentiating Solid pulmonary nodules adenocarcinoma from inflammatory lesions, the PET+CT model performed better than the single-modality models. The RF classifier-based PET+CT model showed high diagnostic discrimination capability and has predictive potential for clinical applications.

## Conclusion

This study demonstrates that ^18^F-FDG PET/CT-based radiomics models may help differentiate pulmonary solitary solid adenocarcinomas from inflammatory nodules. Among the models evaluated, the combination of PET and CT radiomic features yielded superior diagnostic performance compared to single-modality models, with the RF classifier achieving the highest accuracy and stability. These findings suggest that integrating metabolic and anatomical information *via* radiomics can enhance diagnostic precision and support more informed clinical decision-making. Nevertheless, further validation in large-scale, prospective, multicenter studies is warranted to confirm the generalizability and clinical applicability of these models.

##  Supplemental Information

10.7717/peerj.21188/supp-1 Supplemental Information 1 Comparative performance metrics of CT, PET, and PET+CT models

10.7717/peerj.21188/supp-2 Supplemental Information 2 Original data

10.7717/peerj.21188/supp-3 Supplemental Information 3 code

## Additional Information and Declarations

### Data Availability

The following information was supplied regarding data availability: The code and data are available in the Supplemental Files.
