# Supplementary material for: Clinical study of 18F-FDG PET/CT radiomics in differentiating pulmonary solitary solid adenocarcinoma nodules and inflammatory nodules
Source: PeerJ. 2026 Jun 1;14:e21188. doi: 10.7717/peerj.21188 (PMC13235688; doi:10.7717/peerj.21188)
Supplement: Supplemental Information 1 [file peerj-14-21188-s001.docx]

**Table 1. Comparative performance metrics of CT, PET, and PET+CT models**

| Modality | Model | set | AUC | AUC CI Lower | AUC CI Upper | Accuracy | Sensitivity | Specificity | F1-score |
| --- | --- | --- | --- | --- | --- | --- | --- | --- | --- |
| CT | SVM | Training | 0.863 | 0.814 | 0.912 | 0.800 | 0.750 | 0.667 | 0.767 |
|  |  | Validation | 0.807 | 0.729 | 0.884 | 0.716 | 0.839 | 0.750 | 0.655 |
|  | RF | Training | 0.848 | 0.811 | 0.885 | 0.800 | 0.774 | 0.714 | 0.756 |
|  |  | Validation | 0.827 | 0.766 | 0.888 | 0.761 | 0.817 | 0.795 | 0.714 |
|  | SGD | Training | 0.873 | 0.839 | 0.907 | 0.800 | 0.783 | 0.692 | 0.752 |
|  |  | Validation | 0.796 | 0.742 | 0.85 | 0.731 | 0.811 | 0.756 | 0.667 |
|  | KNN | Training | 0.83 | 0.781 | 0.879 | 0.768 | 0.704 | 0.643 | 0.735 |
|  |  | Validation | 0.731 | 0.651 | 0.811 | 0.701 | 0.821 | 0.744 | 0.643 |
|  | XGBoost | Training | 0.813 | 0.761 | 0.865 | 0.794 | 0.762 | 0.700 | 0.75 |
|  |  | Validation | 0.831 | 0.749 | 0.913 | 0.761 | 0.815 | 0.811 | 0.724 |
|  | LightGBM | Training | 0.819 | 0.759 | 0.88 | 0.774 | 0.759 | 0.688 | 0.715 |
|  |  | Validation | 0.814 | 0.715 | 0.913 | 0.761 | 0.784 | 0.829 | 0.733 |
| PET | SVM | Training | 0.756 | 0.728 | 0.784 | 0.729 | 0.667 | 0.708 | 0.687 |
|  |  | Validation | 0.751 | 0.703 | 0.799 | 0.731 | 0.779 | 0.744 | 0.654 |
|  | RF | Training | 0.766 | 0.730 | 0.81 | 0.671 | 0.606 | 0.643 | 0.611 |
|  |  | Validation | 0.81 | 0.748 | 0.873 | 0.701 | 0.719 | 0.744 | 0.643 |
|  | SGD | Training | 0.795 | 0.771 | 0.820 | 0.723 | 0.696 | 0.760 | 0.645 |
|  |  | Validation | 0.807 | 0.77 | 0.843 | 0.776 | 0.737 | 0.786 | 0.717 |
|  | KNN | Training | 0.72 | 0.675 | 0.764 | 0.645 | 0.571 | 0.667 | 0.593 |
|  |  | Validation | 0.792 | 0.712 | 0.872 | 0.716 | 0.706 | 0.750 | 0.655 |
|  | XGBoost | Training | 0.735 | 0.672 | 0.798 | 0.684 | 0.625 | 0.621 | 0.620 |
|  |  | Validation | 0.746 | 0.643 | 0.849 | 0.687 | 0.725 | 0.737 | 0.632 |
|  | LightGBM | Training | 0.782 | 0.733 | 0.832 | 0.729 | 0.689 | 0.643 | 0.667 |
|  |  | Validation | 0.77 | 0.692 | 0.847 | 0.701 | 0.755 | 0.744 | 0.643 |
| PET+CT | SVM | Training | 0.881 | 0.829 | 0.932 | 0.845 | 0.789 | 0.677 | 0.824 |
|  |  | Validation | 0.843 | 0.765 | 0.921 | 0.746 | 0.893 | 0.806 | 0.712 |
|  | RF | Training | 0.849 | 0.799 | 0.892 | 0.781 | 0.746 | 0.750 | 0.734 |
|  |  | Validation | 0.847 | 0.775 | 0.919 | 0.791 | 0.804 | 0.821 | 0.750 |
|  | SGD | Training | 0.891 | 0.856 | 0.926 | 0.832 | 0.820 | 0.667 | 0.794 |
|  |  | Validation | 0.838 | 0.785 | 0.891 | 0.716 | 0.840 | 0.750 | 0.655 |
|  | KNN | Training | 0.833 | 0.777 | 0.888 | 0.761 | 0.694 | 0.679 | 0.730 |
|  |  | Validation | 0.800 | 0.716 | 0.885 | 0.731 | 0.819 | 0.769 | 0.679 |
|  | XGBoost | Training | 0.831 | 0.764 | 0.897 | 0.768 | 0.716 | 0.778 | 0.727 |
|  |  | Validation | 0.843 | 0.742 | 0.945 | 0.806 | 0.807 | 0.825 | 0.764 |
|  | LightGBM | Training | 0.810 | 0.742 | 0.879 | 0.729 | 0.677 | 0.710 | 0.677 |
|  |  | Validation | 0.843 | 0.733 | 0.945 | 0.776 | 0.767 | 0.833 | 0.746 |
